# Editorial: Molecular basis of seed germination and dormancy

**DOI:** 10.3389/fpls.2023.1242428

**Published:** 2023-07-10

**Authors:** Xinyong Liu, Zhoufei Wang, Yong Xiang, Xiaohong Tong, Łukasz Wojtyla, Yifeng Wang

**Affiliations:** ^1^ State Key Laboratory of Rice Biology and Breeding, China National Rice Research Institute, Hangzhou, China; ^2^ The Laboratory of Seed Science and Technology, Guangdong Key Laboratory of Plant Molecular Breeding, State Key Laboratory for Conservation and Utilization of Subtropical Agro-Bioresources, South China Agricultural University, Guangzhou, China; ^3^ Shenzhen Branch, Guangdong Laboratory for Lingnan Modern Agriculture, Genome Analysis Laboratory of the Ministry of Agriculture, Agricultural Genomics Institute at Shenzhen, Chinese Academy of Agricultural Sciences, Shenzhen, China; ^4^ Department of Plant Physiology, Institute of Experimental Biology, Faculty of Biology, Adam Mickiewicz University, Poznań, Poland

**Keywords:** dormancy, hormone crosstalk, seed germination, molecular mechanism, seed physiology, crop improvement

Seeds are both the beginning and the end of the plant life cycle. Plants have evolved complex molecular mechanisms to regulate seed germination to ensure successful seedling establishment under favorable conditions and seed dormancy to allow for seed dispersal in space and time in the natural environment (Bewley et al., 2013). Seed germination occurs in stages that include imbibition, hydration, enzyme activation, cell division, and enlargement. The embryo also breaks through the seed coat and develops into a seed (Godinez-Alvarez et al., 2012; Gu et al., 2012). Additionally, dormancy hinders seed germination through physical, mechanical, or chemical inhibition by the embryo’s outer layers (Bewley et al., 2013). Each stage of germination and dormancy is regulated by a precise molecular mechanism. Previous studies have shown that the mechanism that governs these stages is primarily regulated by the gibberellin (GA) and abscisic acid (ABA) biosynthetic and catabolic pathways (Nambara et al., 2010; Bassel et al., 2011; Dekkers et al., 2013; Finch-Savage and Bassel, 2016). However, the mechanism is still poorly understood. As a result, the title of the current Research Topic, “*Molecular basis of seed germination and dormancy*,” is essential. Seven highly relevant papers were published that explore the molecular basis of different stages of germination and dormancy using various research techniques from different disciplines of biology. Transcriptomics, proteomics, and other field research techniques could help to understand the molecular mechanisms of seed germination and dormancy and promote agricultural application research.

At the same time, light is required to initiate germination in the photodormant seed. In Arabidopsis, the relationship between light and seed germination has been extensively elucidated, showing that phyA and phyB mediate the very low fluence response (VLFR) and the R/FR photo-reversible response (LFR) to seed germination (Seo et al., 2009; Lee et al., 2012; Yang et al., 2020). The molecular mechanism of light-dependent germination differs between photophilic and photophobic seeds. In their work, Liu et al. used tobacco seeds as a model plant to explore the mechanism of seed photodormancy. PAC results of differentially expressed genes (DEGs) and differentially expressed proteins (DEPs) showed that 2S albumin large chain, OLEO1, GAPA1, PSBO1, and PPI form the regulatory network during germination of shallow photo-dormant seeds by (Liu et al.)The molecular regulatory mechanism of light during germination and post-germination development in external photo-dormant tobacco seeds is systematically investigated by integrating transcriptomic and proteomic data.

Previous studies have shown that the balance between ABA and GA significantly regulates seed dormancy and germination (Finch-Savage and Leubner-Metzger, 2006; Holdsworth et al., 2008). ABA signaling dominates the dormant. In contrast, GA signaling is associated with seed germination (Yang et al., 2022; Wang et al., 2023). The Delay of Germination (DOG) gene family plays a vital role in these processes (Graeber et al., 2010; Nonogaki, 2014). Wang et al. employed genomic approaches to compare the effect of different exogenous hormones on recalcitrant seed dormancy. They found that exogenous abscisic acid can inhibit embryonic development and delay germination in the resistant seed of *Panax notoginseng*. In addition, the molecular mechanism is analogous at the transcriptional level. Wang et al. found that pyrabactin resistance-like (PYL), SNF1-related protein kinase subfamily 2 (SnRK2s), and type 2C protein phosphatase (PP2C) increase ABA and suppress GA signaling in ABA-treated *P*. *notoginseng* seeds. They discovered that MAPK signaling cascades may play an essential role in hormone signal transmission.

In seed heteromorphism, a single plant produces two or more diaspores with different specializations in dispersal mechanisms, germination characteristics, and dormancy levels (Abdul Aziz et al., 2021; Gianella et al., 2021). Loades et al. analyzed the dimorphic seeds (black and brown) of the amaranthaceous weed *Chenopodium album* for dormancy and germination. They observed that the non-dormant brown seed has a thinner testa, higher endogenous GA, and faster degradation of ABA during imbibition compared to the black seed. The 14 genes covering major subgroups of GA and ABA metabolism, GAox20, GAox3, NCED, and CYP707A, were identified by (Loades et al.)

It is well known that seed germination and dormancy are regulated by a network of transcription factors (Roschzttardtz et al., 2009). In a previous study, three B3 domain factors (LEAFY COTYLEDON2 [LEC2], FUSCA3 [FUS3], and ABSCISIC ACID INSENSITIVE3 [ABI3]) and LEC1 (a HEME-ACTIVATED PROTEIN3 subunit of CCAAT binding factors) were identified as key regulators of zygotic embryo development in Arabidopsis (Lotan et al., 1998; Stone et al., 2001; Baumbusch et al., 2004; Wang and Perry, 2013). LEC1 and FUS3 play an essential role in viviparous germination and embryonic development in the mangrove Kandelia obovata, according to findings published by Zhou et al. They used the Weighted Gene Co-expression Network Analysis (WGCNA) method to analyze the overall expression network and discovered 297 Arabidopsis phytohormone genes involved in catabolism, biosynthesis, and signal transduction of ABA, GA, BR, cytokinin, auxin, and ethylene, in addition to six homologs, namely *LEC1*, *ABF-8*, *SAM-1*, *FUS3-2*, *CYP707A-3*, and *BAS1-3*. Interestingly, they also found that photosynthesis-related pathways were significantly up-regulated in viviparous embryos and that substance transporter genes were highly expressed in the seed coat. This array of genes may bridge the relationship between the viviparous phenomenon and the pre-harvest germination phenomenon.

Seed germination is controlled by numerous biological processes, namely membrane, DNA, and mitochondrial repair, in which gene expression and protein synthesis are involved (Zhang et al., 2016). Alternative splicing (AS) can generate multiple mRNA variants from a single gene, greatly expanding the coding ability of the genome (James et al., 2012; Feng et al., 2015; Chen et al., 2019). Sybilska et al. reviewed the alternative splicing of ABA signaling-related genes during seed germination. They present a recent study on the identified AS regulators and the ABA-related changes in AS during seed germination. They described the alternative splicing of *HAB1*, *ABI3*, *VP1*, *PIF6*, *DOG1*, etc. For instance, HYPERSENSITIVE TO ABA1 (HAB1), which inhibits ABA signal transduction, has two AS isoforms. The HAB1.1 isoform promotes germination, whereas the HAB1.2 isoform inhibits germination (Finkelstein et al., 2008; Nakabayashi et al., 2012; Wang et al., 2015; Dekkers et al., 2016). This review has further deepened our understanding of alternative splicing during seed germination.

Overall, this Research Topic provides deeper insights into the molecular basis of seed germination and dormancy, highlighting some of the important molecular mechanisms contributing to seed germination and dormancy. The research of Liu et al. and Zhou et al. systematically analyzed the regulatory mechanism of light during germination and the long-standing mystery of the vivipary mechanism by integrating transcriptomics and proteomics. Wang et al. provided a novel insight into the dormancy regulation of recalcitrant seeds, which may contribute to their storage. Loades et al. revealed the dormancy difference of the dimorphic seeds and provided a bet-hedging strategy in weeding. We hope that this Research Topic on the molecular basis of seed germination and dormancy will be an important reference for broader seed biology and provide theoretical support for production applications in the future.

## Author contributions

All the authors participated in the editing of this Research Topic. XL wrote the draft, and all the other authors provided suggestive comments on the editorial. All authors contributed to the article and approved the submitted version.
